# *RpoZ* regulates 2,4-DAPG production and quorum sensing system in *Pseudomonas fluorescens* 2P24

**DOI:** 10.3389/fmicb.2023.1160913

**Published:** 2023-05-12

**Authors:** Yarui Wei, Baozhu Dong, Xiaogang Wu, Mingmin Zhao, Dong Wang, Na Li, Qian Zhang, Liqun Zhang, Hongyou Zhou

**Affiliations:** ^1^College of Horticulture and Plant Protection, Inner Mongolia Agricultural University, Hohhot, Inner Mongolia, China; ^2^College of Agriculture, Guangxi University, Nanning, China; ^3^Erdos Agricultural and Animal Husbandry Technology Promotion Center, Erdos, Inner Mongolia, China; ^4^Bayannaoer Agriculture and Animal Husbandry Technology Promotion Center, Bayannaoer, Inner Mongolia, China; ^5^Department of Plant Pathology, China Agricultural University, Beijing, China

**Keywords:** *Pseudomonas fluorescen* 2P24, *rpoZ*, 2,4-DAPG, quorum sensing, biocontrol

## Abstract

**Introduction:**

*Pseudomonas fluorescens* 2P24 was isolated from soil of natural decay associated with wheat take-all and it can effectively control soil-borne diseases caused by a variety of plant pathogens. 2,4-diacetylphloroglucinol (2,4-DAPG), is produced by *P. fluorescens* 2P24 and plays an important role in the prevention and control of plant diseases. To understand the resistant mechanism, in this study, we conducted experiments to explore the regulation role of *rpoZ* in the synthesis of the antibiotic 2,4-DAPG and regulation of QS system.

**Methods:**

A random mini-Tn5 mutagenesis procedure was used to screen regulators for *phlA* transcription in stain PM901, which containing a phlA∷lacZ transcriptional fusion reporter plasmid. We identified 12 insertion mutants could significantly change *phlA* gene expression. By analyzing the amino acid sequences of the interrupted gene, we obtained a mutant strain Aa4-29 destroyed the *rpoZ* gene, which encodes the omiga subunit. We constructed the plasmid of *rpoZ* mutant (pBBR-△rpoZ) transformed into competent cells of *P. fluorescens* 2P24 by electro-transformation assay. The strains of *P. fluorescens* 2P24/pBBR, 2P24-△rpoZ/pBBR, 2P24-△rpoZ/pBBR-rpoZ were used to evaluate the regulation role of *rpoZ* in 2,4-DAPG production and quorum sensing system.

**Results:**

According to β-galactosidase activity, we found that *rpoZ* positively regulated the expression of *phlA* (a synthesis gene of 2,4-DAPG) and *PcoI* (a synthesis gene of PcoI/PcoR QS signal system) at the transcriptional level. The production of 2,4-DAPG antibiotic and signal molecule AHL was influenced by *rpoZ*. Further, *rpoZ* was involved in regulating *rsmA* expression. *RpoZ* also has a certain regulatory effect on *rpoS* transcription, but no effect on the transcription of *phlF*, *emhABC* and *emhR*. According to the biocontrol assay, *P. fluorescens* 2P24 strains with *rpoZ* showed obvious antagonism ability against the *Rhizoctonia solani* in cotton, while the mutant strain of *rpoZ* lost the biocontrol effect. *RpoZ* had a significant effect on the swimming and biofilm formation in *P. fluorescens* 2P24.

**Conclusion:**

Our data showed that *rpoZ* was an important regulator of QS system, 2,4-DAPG in *P. fluorescens* 2P24. This may imply that *P. fluorescens 2P24* has evolved different regulatory features to adapt to different environmental threats.

## 1. Introduction

Plant growth-promoting rhizobacteria (PGPR) are closely related to plant roots, which can affect plant health and soil fertility. *Pseudomonas fluorescens* 2P24 generally colonizes in the root, inhibits plant soil borne diseases through producing 2,4-diacetylphloroglucinol (2,4-DAPG; Wei et al., 2004). The biosynthetic pathway of 2,4-DAPG has been clarified in several *Pseudomonas* strains (Vincent et al., 1991; Fenton et al., 1992). The 2,4-DAPG locus includes the four biosynthetic genes *phlACBD* that are transcribed as a single operon and is directly involved in the catalytic process of 2,4-DAPG production (Bangera and Thomashow, 1999). Among of them, *PhlA*, *phlC* and *phlB* are required for transacetylation of the monoacetylphloroglucinol (MAPG) precursor to generate DAPG and *phlD* is critical for the biosynthesis of MAPG (Bangera and Thomashow, 1999; Schnider-Keel et al., 2000). The *phlF* gene, which encodes a TetR-family transcriptional regulator, is located upstream of the *phlA* gene and blocks *phlACBD* transcription by binding to the *phlA* promoter region (Schnider-Keel et al., 2000; Li et al., 2018). Whereas, *phlG,* a gene located between the *phlF* and *phlH* genes (upstream of the *phlACBD* biosynthetic operon), mediates the conversion of DAPG to MAPG in *P. fluorescens* 2P24 (Zhao et al., 2020).

Except for the antibiotic production of *P. fluorescens* 2P24, another factor, quorum sensing (QS) regulation, should also pay attention in bacteria. QS system plays an important role in a diverse array of physiological activities, including symbiosis, virulence, competence, conjugation, antibiotic production, swarming, sporulation and biofilm formation (Kievit and Iglewski, 2000; Gonzalez and Keshavan, 2006; Wei and Zhang, 2006; Sakuragi and Kolter, 2007; Waters et al., 2008). The discovery of new regulators of QS system will help to further elucidate the signal transduction mechanism that bacteria survive under various environmental conditions. In *Pseudomonas*, regulatory elements of QS system, the stationary-phase sigma factor RpoS (Bertani and Venturi, 2004), the two-component regulatory system GacS/GacA (Reimmann et al., 1997), the small RNA-binding regulator RsmA (Pessi et al., 2001), the LuxR family member VqsR (Juhas et al., 2004) and the tetrahelical H-T-H superclass member RsaL (Rampioni et al., 2007) were identified. In *P. fluorescens* 2P24, the *GacS-GacA* system controls its target *phlACBD* by inducing four sRNAs (*RsmX*, *RsmX1*, *RsmY*, and *RmZ*) and repressing the levels of another sRNA, *RgsA* (Zhang et al., 2020a). The RsmA and RsmE proteins directly repress the translation of *phlACBD mRNA*, whereas four sRNAs (*RsmX*, *RsmX1*, *RsmY*, and *RsmZ*) depress the translation of *phlACBD* mRNA by sequestering the *RsmA* and *RsmE* proteins, thereby inducing the production of 2,4-DAPG (Zhang et al., 2020a,b). It was also reported that a quorum-sensing locus, *pcoI*/*pcoR*, which is involved in the regulation of root colonization and plant disease-suppressive ability in *P. fluorescens* 2P24, (Yan et al., 2009a).

DNA-dependent RNA polymerase (RNAP) is the central enzyme involved in gene expression and also constitutes a major target for genetic regulation (Darst et al., 1989, 1991; Schultz et al., 1993). The bacterial RNAP core enzyme consists of four subunits: alpha (α), beta (β), beta’ (β’) and omega (ω) subunit (Lonetto et al., 1992; Zhang et al., 1999). Among of them, the ω subunit encoded by the *rpoZ* gene was proposed to be an integral part of the core RNAP and is not essential for RNAP activity and cell survival, but can assist RNA polymerase assembly, help β ‘subunits fold and protect β’ subunits (Burgess, 1969; Dove and Hochschild, 1998; Ghosh et al., 2001; Mathew and Chatterji, 2006). *Streptomyces kasugaensis* produces an antibiotic called primathromycin (KSM), an aminoglycoside antibiotic, to control *Pyricularia oryzae* in rice. Kojima et al. showed that the production of primathromycin in the *rpoZ* mutant strain was reduced, and the formation of aerogenic mycelia was blocked, and these phenotypes could be restored to the wild type by the transfer of *rpoZ* complementary plasmid into the mutant strain (Kojima et al., 2002). This is indicating that *rpoZ* in *S. primaviae* regulated the production of primavithromycin. Transcription analysis of KSM synthesis genes showed that the expression of *kasT* (a specific transcriptional activator synthesized by KSM) was significantly decreased in *rpoZ* mutants, and the expression of *kasT* may require the participation of *rpoZ* or RNAP (including ω subunits). When the *rpoZ* gene of *Mycobacterium smegmatis* was mutated, the colony morphology of the *rpoZ* mutant strain was changed, and swimming ability, the biofilm formation, the strain cell growth were affected as well (Mathew et al., 2006).

As we have discussed above, *P. fluorescens* and its secondary metabolites play a very important role in biocontrol strategies. In this study, we identified a regulator of *rpoZ* in *P. fluorescens* 2P24, which is similar to *rpoZ* of several bacteria. The results showed that *rpoZ* regulates several genes including *phlA*, *pcoI*, *rpoS*, which indicated it might be an important upstream regulator of QS in *P. fluorescens* 2P24. We also found that strains with *rpoZ* showed obvious antagonism ability against the *Rhizoctonia solani* in cotton, and had significant effect on the swimming and biofilm formation in *P. fluorescens* 2P24.

## 2. Materials and methods

### 2.1. Bacterial strains and growth condition

Bacterial strains and plasmids used in this study are listed in Supplementary Table S1. *Escherichia coli* and *P. fluorescens* were cultured as described previously in (Wu et al., 2012; Zhao et al., 2020). *E. coli* was grown in Lysogenic broth (LB) medium at 37°C and *P. fluorescens* strains were grown at 28°C in LB medium, KB (King’s B medium; King et al., 1954) or ABM medium (Chilton et al., 1974).

### 2.2. Construction of *rpoZ* mutant and complementation strain

According to the flanking sequence of *rpoZ* gene of strain *P. fluorescens* 2P24, two pairs of primers, rpoZ 29,729/rpoZ 30,442 and rpoZ 30,518/rpoZ 31,424, were designed to amplify *rpoZ* gene (Supplementary Table S2). Using the genome of wild bacterium 2P24 as template, the upstream and downstream *rpoZ* genes were amplified with the length of 907 bp and 714 bp, respectively. The PCR products were treated with the restriction enzymes, *EcoR* I/*Kpn* I and *Kpn*I/*Hind* III respectively, and then cloned into the vector pBLR digested with the corresponding *EcoR*I/*Hind* III restriction enzyme to obtain the suicide vector pBLR- △rpoZ. The construct was verified by diagnostic PCR by primer pair of G1/G2 and Ga/Gd using 2P24 genomic DNA and plasmid p299△G. A 378 bp fragment was lost in p299△G comparing with the wild type *rpoZ* gene according to the confirmation PCR and sequencing.

The plasmids of pBLR-△rpoZ were transformed into competent cells of *P. fluorescens* 2P24 by electro-transformation assay under screening of Km resistance in LB liquid medium. After 7 generations, bacteria grown on ABM medium containing Km and X-gal under the condition of 28°C for 24 h. The white clones were verified by PCR amplification.

The *rpoZ* gene was cloned into the shuttle vector pBBR, which was used for complementary experiments. Using the genome DNA of *P. fluorescens* 2P24 as template, primers rpoZ 30,059/rpoZ 30,712 were used to obtain the fragment of *rpoZ* gene. The fragment was digested by *Hind* III - *Kpn*I and connected with pBBR to obtain the complementary vector, named as plasmid pBBR-rpoZ.

In order to amplify the complete rpoZ gene, PCR primers rpoZ 29,729/rpoZ 30,442 and rpoZ 30,518/rpoZ 31,424 were designed based on the gene sequence of *Pseudomonas fluorescens* 2P24. Using *P. fluorescens* 2P24 genome as a template, two amplified fragments were digested by EcoRI/KpnI and KpnI/HindIII restriction endonuclease enzyme, respectively, and then linked to the corresponding enzyme digested vector pBLR, and a suicidal deletion vector pBLR-△rpoZ was obtained. Using PCR primer rpoZ 30,059/rpoZ 30,712 and wild bacterium 2P24 as template, the 654 bp target fragment containing the complete rpoZ gene was amplified and then connected to the shuttle vector pBBR after being digested by Hind-III/Kpn I restriction enzyme. The complementary vector pBBR-rpoZ was obtained. The deletion vector and complementary vector were transferred into wild-type 2P24 strain to obtain the deletion mutant strain and complementary strain of rpoZ (Supplementary Figures S1A,B).

Each strain was cultured in ABM and LB liquid medium overnight, and the culture concentration was adjusted to the same OD_600_ = 0.8 with ABM and LB liquid medium, and then transferred to 40 ml ABM and LB liquid medium containing corresponding Amp and Gm antibiotics at a ratio of 1:1000, respectively. Then, placed in a 28°C incubator for shaking with120 r/min, and collected samples and measured OD_600_ every 3 h. Each sample was repeated 3 times.

### 2.3. Detection of QS signal molecule (AHL) in *Pseudomonas fluorescens* 2P24 and its derived strains

Strains of *P. fluorescens* 2P24/pBBR, 2P24-△rpoZ/pBBR, 2P24-△rpoZ/pBBR-rpoZ were inoculated into 5 ml LB medium containing Amp and Gm antibiotics, placed in a shaker with 120 r/min for 36 h at 28°C. 30 μl was inoculated into 30 ml LB medium containing Amp and Gm antibiotics and placed in a shaker for 120 r/min at 28°C. 800 μl of each bacterial solution to be tested was added into the same volume of ethyl acetate, and signals were extracted by extraction method. The organic phase was air-dried and dissolved in 100 μl methanol, diluted 10 times, and stored at −20°C. The reported strain *A. tumefaciens* NTL4 (pZLR4) was inoculated into ABM liquid medium, incubated in a shaker with 120 r/min for 24 h at 28°C, and stored at 4°C. 200 μl newly cultured reporter bacteria (*A. tumefaciens* NTL4) was measured after being cultured with 5 μl of each signal molecule for 4 h. Reporter bacteria added with 5 μl methanol were used as the control.

The QS signals were detected by β-galactosidase activities as described previously in (Miller, 1972). All experiments were performed in triplicate (Wu et al., 2012). Each strain was set 3 replicates.

### 2.4. Determination of 2,4 DAPG production

Quantification of 2,4-DAPG was done as described previously (Shanahan et al., 1992). *P. fluorescens* 2P24/pBBR, 2P24-△rpoZ/pBBR, 2P24-△rpoZ/pBBR-rpoZ was inoculated into 40 ml King’s B liquid medium and incubated in a shaker at 28°C with 120 r/min for 40 h to stationary stage. After centrifugation at 8,000 r/min for 10 min, the supernatant was taken out and acidified to pH 2.0 with 1 mol/l HC1. Equal volume of ethyl acetate was added for extraction, and the organic phase was extracted by rotary evaporation. The dry matter was dissolved with 50 ml methanol and determined by HPLC (UV2002 high performance Liquid chromatograph). C_18_ reverse column was used for HPLC: diameter: 150 × 4.6 mm; Detection wavelength: 270 nm; sample volume: 5 μl; mobile phase: water: ethyleye (V: V) = 45: 55, 0.1% H3P04; Flow rate: l.0 ml/min; retention time: 5.12 min. 2,4-DAPG chromatographic standard sample was purchased from Toronoto Research Chemicals Inc., (D365500).

### 2.5. Antagonism test of *rpoZ* in *Pseudomonas fluorescens* 2P24 against *Rhizoctonia solani*

Cultivation of *Rhizoctonia solani* and dual-culture confrontation assay was performed on PDA medium (Zhao et al., 2020). The fungus disk of strains with a diameter of 6 mm was placed in the center of the PDA plate, and the tested biocontrol bacteria were inoculated 2.5 cm away from the fungus disk. The plates were placed in an incubator at 25°C. The size of the inhibition zone was measured when the control fungus grew to the edge of the petri dish. Three replicates per treatment.

### 2.6. Motility tests

Motility tests were conducted as described by Rashid and Kornberg (2000), with slight modification (Wang et al., 2021). For swimming tests, we used water medium that contained 0.2% agar. Freshly cultured strains were dipped using pipette tips and inoculated on the surface of the center of plates. Then, plates were placed stably in the incubator and cultured at 28°C. After 24 h, the diameter of motility of clones was measured.

### 2.7. The biofilm formation of *Pseudomonas fluorescens* 2P24 and its derived strains

To test whether *rpoZ* affect the biofilm formation of *P. fluorescens* 2P24, the quantitative determination of biofilms is performed as described (Wei and Zhang, 2006) with slight modification. The strain of *ropZ* mutant was inoculated into LB liquid medium and cultured until saturated, then diluted into fresh LB liquid medium at the volume ratio of 1:1000. The formation of bioflim at the junction of solid and liquid was measured after 500 μl was added into a 2 ml centrifugation tube and incubated at 28°C for 24 h. Add 100 μl crystal violet with a concentration of 0.1% (W/V) to each tube. After 20–30 min staining at room temperature, rinse the centrifuge tube with strong distilled water. In the inside of the centrifuge tube, it can be observed to form a strong biofilm at the junction of the liquid level and the tube wall. The crystal violet combined with biofilm was fully dissolved by adding 1,200 μl of 95% ethanol, and the absorption value of OD_570_ was measured with 1,000 μl.

### 2.8. Statistical analysis

GraphPad Prism software version 5.01 (Graphpad Software, Inc.) was used for analysis of variance, followed by multiple comparisons using one way ANOVA, and *p* < 0.05 was considered statistically significant.

## 3. Results

### 3.1. Identification and characteristic of *rpoZ* gene in *Pseudomonas fluorescens* 2P24

**Figure 1:**
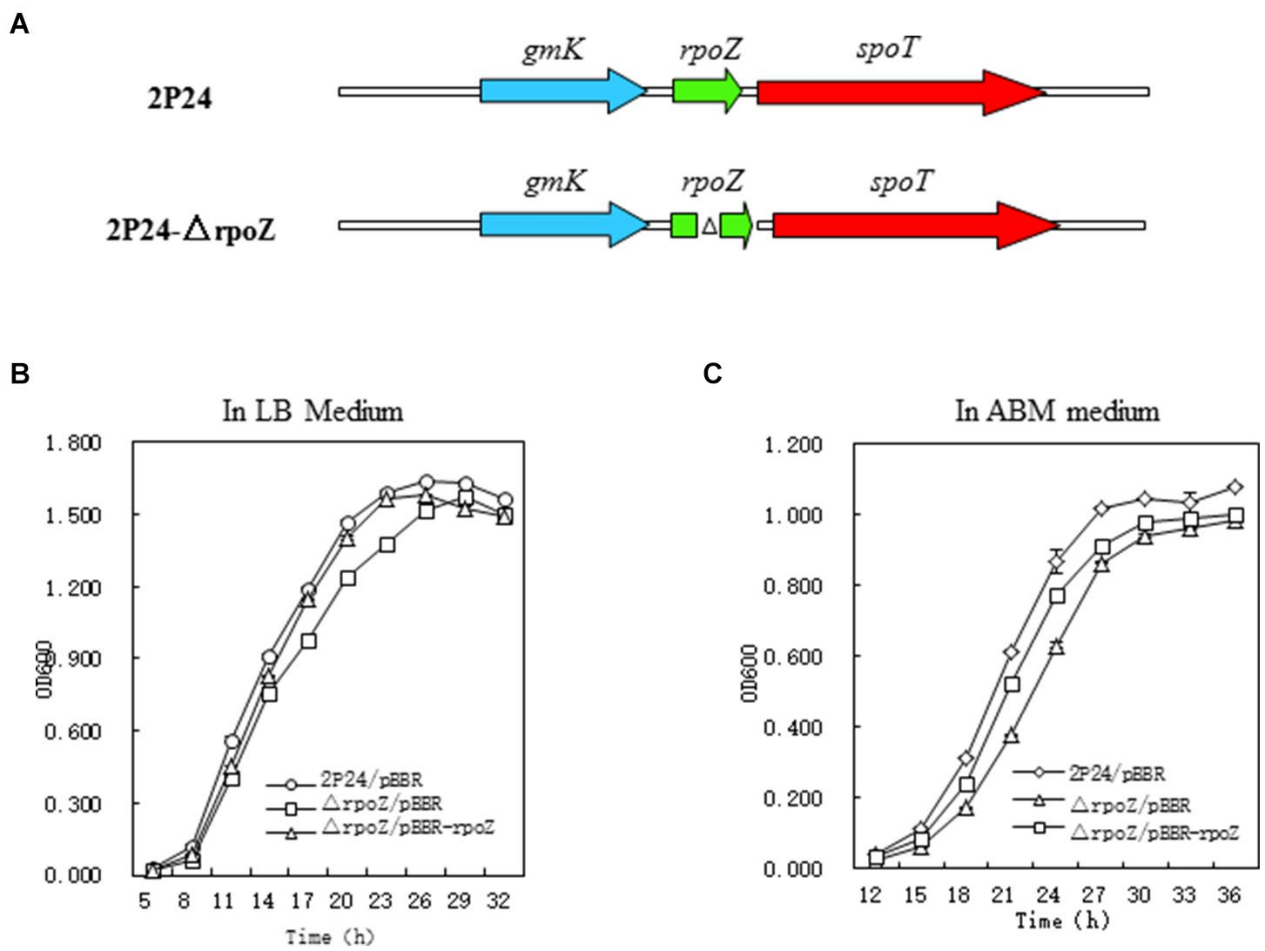
Structure and growth curve of rpoZ gene mutation. **(A)** Schematic diagram of the construction of the *ropZ* deletion mutant in *Pseudomonas fluorescens* 2P24. **(B,C)** Growth rate of wild-type (WT) *P. fluorescens* 2P24 and the *ropZ* deletion mutant in LB and ABM liquid medium. The error bars represent standard deviations and the statistical analysis was performed using a two-tailed *t*-test. Three replicates per treatment.

The random insertion method of Tn5 transposon was used to mutate the transcriptional fusion plasmid pGm-phlA contained in *P. fluorecens* 2P24 and screened the regulatory factors affecting the production of 2,4-DAPG. We identified 21 mutant strains that significantly reduced *phlA* expression (Supplementary Table S3). Because of Tn5 transposon is carrying Gm antibiotic resistance gene, the genes damaged by Tn5 transposons in mutant strains could be determined by analysis of the flanking sequence of Tn5. Restriction enzymes (*Sal*I/*EcoR*I) were used for enzyme digestion and self-ligated. Plasmids were transformed into *E. coli* DH5α and inoculated on LB solid medium containing 30 μg/ml antibiotics Gm. The single colony containing the flanking sequence of Tn5 transposon was selected and examined by sequencing. The mutant Aa4-29 with the most reduced *phlA* gene expression was purified and further investigated. Sequence analysis showed that Tn5 was inserted into *rpoZ* gene of mutant strain Aa4-29, which was named as 2P24-△rpoZ (Figure 1A). We constructed the deletion mutant and complementary strain of *rpoZ* as shown in Supplementary Figures S1A,B.

This gene is the ω-subunit of the encoding RNA polymerase, which encodes the synthesis of a 90 amino acid peptide chain with a molecular weight of about 10,105 Da. The RpoZ protein sequence in strain 2P24 is very similar to that of other RpoZ in *Pseudomonas*, among which has a similarity of 90% with the RpoZ sequence in *Pseudomonas brassicacearum* NFM421, has a similarity of 93% with the RpoZ sequence of *Pseudomonas fluorescens* PF0-1 and has a similarity of 86% with the RpoZ sequence of *Pseudomonas syringae* pv.*tomato* str.DC3000.

Analysis of the laterals of the Tn5 transposon revealed that there was a *Gmk* gene in the same transcriptional direction upstream of the *rpoZ* gene, which reportedly encodes the guanylate kinase (Supplementary Figure S1A). The downstream of the *rpoZ* gene is *spoT* gene, whose transcription direction is the same as *rpoZ*. *SpoT* gene encodes pyrophosphatase and is involved in regulating (p)ppGpp level in cells. The sequences of these three genes are also much conserved to upstream and downstream genes of *rpoZ* in *E. coli* and *Streptomyces cerulosus* (Santos-Beneit et al., 2011).

To test the effect of *rpoZ* mutant strain on the growth of *P. fluoresens* 2P24, we compared the wild-type strain *P. fluoresens* 2P24, rpoZ-deficient mutant strain △rpoZ/pBBR and complementary strain △rpoZ/PBBR-rpoZ in LB and ABM medium. The growth curve was drawn according to OD_600_ value at each time point (Figure 1C). The results showed that the growth curves of *P. fluorescens* 2P24/pBBR, △rpoZ/pBBR and △rpoZ/PBBR-rpoZ in LB and ABA medium were consistent (Figure 1B). The growth rate of the *rpoZ* mutant strain was significantly slower than that of the wild-type strain. This growth of the complementary strain carrying the rpoZ plasmid was recovered. These results indicated that the *rpoZ* gene played a role in the regulation of bacterial growth.

### 3.2. *RpoZ* regulates transcription of signal synthesis *pcoI* gene and production of signal molecule AHL

**Figure 2:**
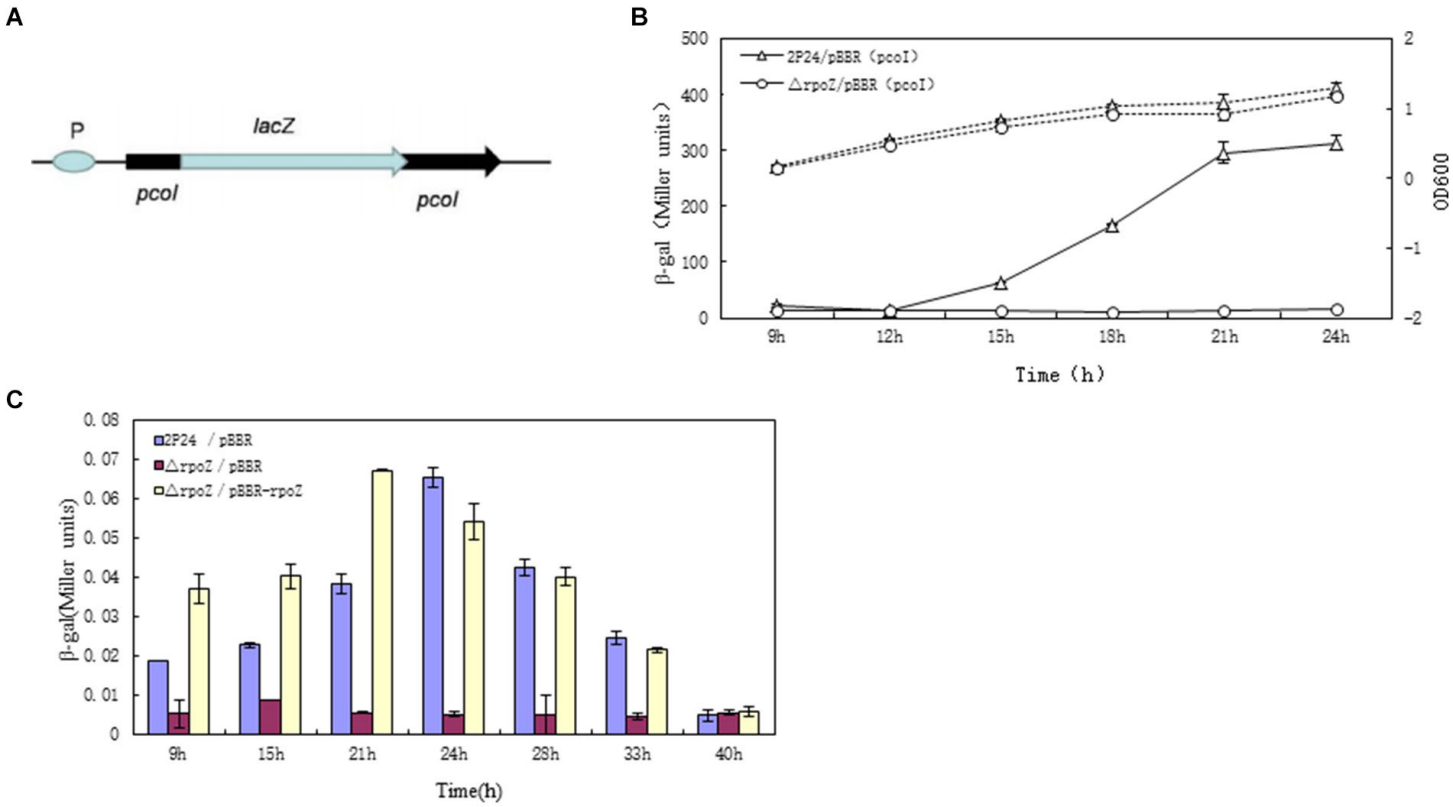
*RopZ* regulates *pcoI* expression in *P. fluorescens* 2P24. **(A)** The graphic presentation of constructed *E. coli* reporter plasmids. pSB-pPcoI contained the promoter region of *pcol* fused to the promoterless *lacZ* gene. **(B)** The β-galactosidase activity of wild type 2P24/pBBR (pcoI) and ropZ mutant △rpoZ/pBBR (pcoI) was detected and shown as the solid lines with Miller units. The dotted lines were growth curve. **(C)** Detection of the signal accumulation of ropZ mutants in *A. tumefaciens* NTL4 (pZLR4). The signal molecules were extracted from wild type 2P24/pBBR, ropZ mutant △rpoZ/pBBR and complementary strain △rpoZ/pBBR-rpoZ cultures and incubated with *A. tumefaciens* NTL4 (pZLR4). Then, the β-gal activities were detected at different time points as shown in horizontal coordinate. The ordinate represents the ratio of β-galactosidase activity to OD_600_ value of each detected strain. Each value was calculated by 3 replicates. The values were from at least three independent assays. Three independent experiments were performed and the error bars were calculated standard deviations of experimental data.

A promoter-free *lacZ* gene into the *pcoI* gene of the QS system on the genome of wild-type strain 2P24 was inserted and constructed pcoI∷lacZ as a marker gene for transcription fusion previously by (Yan et al., 2009b). The transcription of *lacZ* was driven by the *pcoI* gene promoter, and its schematic structure was shown in Figure 2A, by which we determined the level of *pcoI* gene transcription by detecting the β-galactosidase (LacZ) activity of the strain.

The β-galactosidase activity of wild-type strain of *P. fluorescens* 2P24/pBBR and *rpoZ* gene mutant △rpoZ/pBBR was measured and the growth curve was drawn by the value of OD_600_ in the culture medium (Figure 2B). The growth curve showed that the mutant *rpoZ* gene delayed the growth of the bacteria compared with the wild-type strain. The β-galactosidase activity of △rpoZ/pBBR was significantly lower than that of wild-type strain 2P24/pBBR during the whole growth process, which indicated that the transcription activity of *pcoI* gene was greatly reduced after *rpoZ* gene mutation. We suggested that *rpoZ* positively regulated the expression of *pcoI* gene in strain *P. fluoresens* 2P24 at the transcriptional level.

In *P. fluoresens* 2P24, the *pcoI* gene is a synthase that is responsible for synthesizing quorum sensing signalling molecules. We also confirmed *rpoZ* gene also affects the synthesis of signal molecules in the QS system through regulation of *pcoI* expression. The signal molecules produced by the wild strains and the derived strains were extracted and interacted with the reported strain *A. tumefaciens* NTL4 (pZLR4). NTL4 is a modified engineering strain that cannot generate signal molecule itself, but it carries TraG∷LacZ fusion gene to detect exogenous QS signal molecule and promote the expression of LacZ (Chai et al., 2001). Therefore, the LacZ activity level of strain *A. tumefaciens* NTL4 can be reported to compare the content of signal molecules in each sample to be tested.

Compared with the wild strain 2P24, the β-galactosidase activity of the reported strain was significantly decreased by the extract of the signal molecule of △rpoZ/pBBR, which meant that the production of signal molecule of △rpoZ/pBBR was smaller than that of wild type 2P24. And this change can be restored by the intact *rpoZ* gene carried by the plasmid PBBR-rpoZ. Therefore, we believed that *rpoZ* gene positively regulated the synthesis of signal molecules in *P. fluoresens* 2P24. This was consistent with the positive regulation of *pcoI* gene transcription by *rpoZ*, which was proved that *rpoZ* played a positive role in quorum sensing system.

In addition, the signal molecules produced by wild-type strain 2P24 have obvious characteristics on different culture time points (Figure 2C). In the early stage of culture, the synthesis amount of signal molecules was very low. However, with the increase of culture time, the accumulation of signal molecules reached the peak at 24 h, and after the highest accumulation maintained for a period of time, the content of signal molecules in the culture medium began to decline again.

### 3.3. Effects of *rpoZ* gene on transcription of antibiotic synthesis gene *phlA* and 2,4-DAPG production

**Figure 3:**
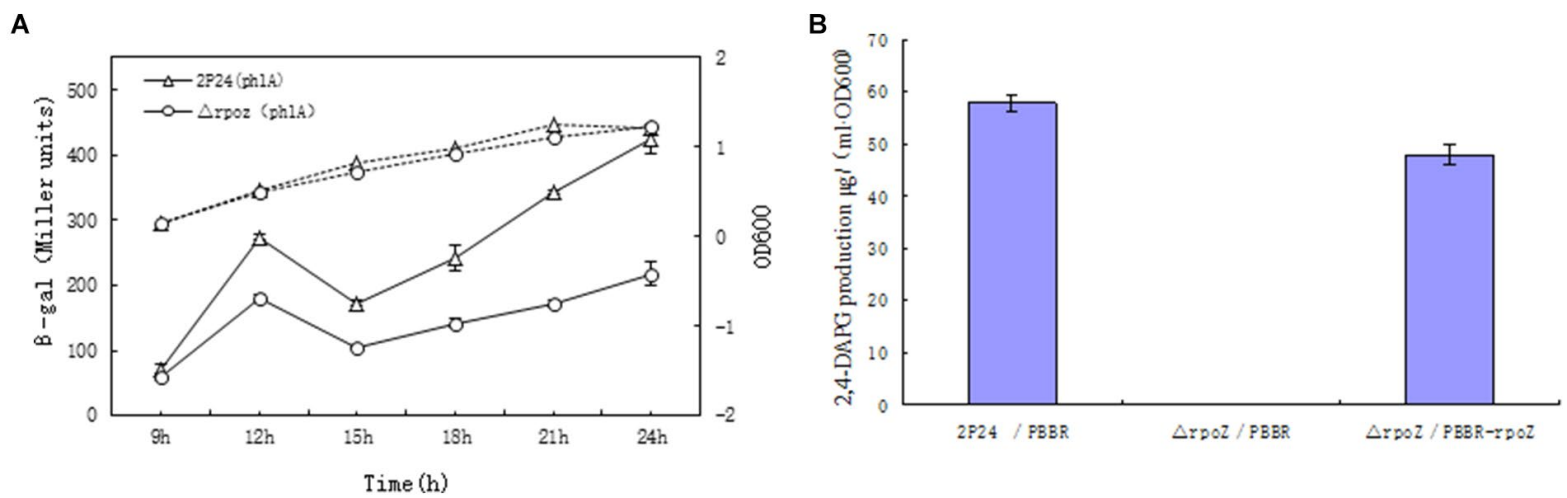
*RopZ* regulates *phlA* expression and 2,4-DAPG production in *P. fluorescens* 2P24. **(A)** β-galactosidase activity of wild type 2P24 and △rpoZ and ropZ mutant was detected and shown. **(B)** 2,4-DAPG production was detected in from wild type 2P24/pBBR, ropZ mutant △rpoZ/pBBR and complementary strain △rpoZ/pBBR-rpoZ.

It was reported that extracellular secondary metabolite 2,4-DAPG from *P. fluoresens* 2P24 played an important role in controlling effects on soil borne disease in wheat (Zhou et al., 2005). The 2,4-DAPG production was regulated by antibiotic synthesis gene *phlA*. To investigate whether *rpoZ* gene ws involved in transcription of *phlA* and 2,4-DAPG production, the activity of β-galactosidase in PhlA-Lacz transcription fusion was detected in wild-type strain 2P24 and △rpoZ/pBBR. The β-galactosidase activity of each strain was measured and the growth curve was drawn by the value of OD_600_. The results showed that the growth of the bacteria containing the mutant *rpoZ* gene significantly delayed compared with that of the wild-type strain (Figure 3A). The expression of *phlA* gene in △rpoZ/pBBR was significantly lower than that of wild-type strain 2P24/pBBR during the whole growth process, which indicated that the transcription activity of *phlA* gene was greatly reduced in △rpoZ/pBBR. This was suggested that *rpoZ* positively regulated the expression of *phlA* gene in strain *P. fluoresens* 2P24 at the transcriptional level.

Correspondingly, HPLC was used to detect whether the yield of 2,4-DAPG was consistent with the positive regulatory effect of *rpoZ* gene on the expression of *phlA* gene. The 2,4-DAPG production of △rpoZ/pBBR was minimal compared with that of wild strain 2P24, and this change was recovered by the intact *rpoZ* gene carried by the plasmid △rpoZ/pBBR (Figure 3B). Therefore, we believed that *rpoZ* gene in *P. fluoresens* 2P24 has an effect on the production of 2,4-DAPG antibiotics.

### 3.4. *RpoZ* affects 2,4 DAPG production through positive regulation of *rsmA*

**Figure 4:**
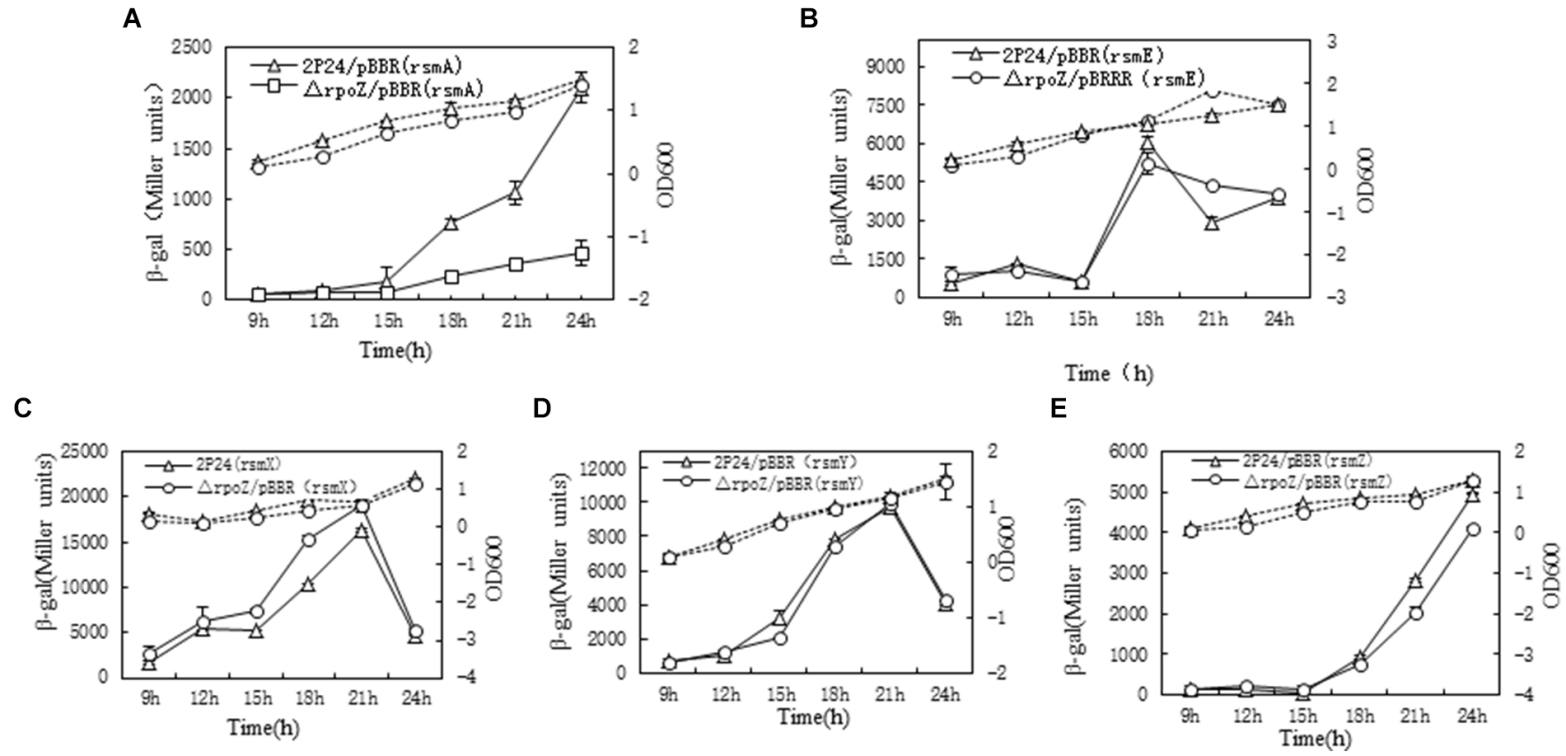
Determination of rpoZ gene influence gene related with 2,4 DAPG production. To determine ropZ regulation effect on expression of rsmA, rsmE, rsmX, rsmY and rsmZ, the β-gal activities in different strains were detected at different time points as shown in **A–E**. The β-galactosidase activity to OD_600_ value of each detected strain were shown as the solid lines with Miller units. The dotted lines were growth curve. Each value was calculated by 3 replicates. The values were from at least three independent assays. Three independent experiments were performed and the error bars were calculated.

In *P. fluorescens* 2P24, the RsmA and RsmE proteins directly repress the translation of phlACBD mRNA, whereas four sRNAs RsmX, RsmX1, RsmY, and RsmZ derepress the translation of phlACBD by sequestering the RsmA and RsmE proteins, thereby inducing the production of 2,4-DAPG (Zhang et al., 2020b). To examine how influence of *rpoZ* on those genes of *rsmA*,*rsmE*, three sRNAs *rsmX*, *rsmY*, and *rsmZ*, the different transcription LacZ fusion reporter plasmids of small RNA molecules (rsmX-lacZ, rsmY-LacZ, rsmZ-lacZ) were constructed and electrocuted into wild bacteria *P. fluorescens* 2P24/pBBR and △rpoZ/pBBR. The β-galactosidase activity of each bacterium was measured and profiled as shown in Figure 4.

The results showed that *rpoZ* gene has no effect on the transcription expression of *rsmE* gene, but *rpoZ* gene has a significant effect on the transcription expression of *rsmA* (about 4 times) (Figures 4A,B). *rpoZ* gene mutation had no effect on the transcription of *rsmX*, *rsmY* and *rsmZ* genes, which indicated that *rpoZ* was not involved in the expression of r*smX*, *rsmY* and *rsmZ* genes at the transcription level in strain 2P24 (Figures 4C–E). *RsmX*, *rsmY* and *rsmZ* genes are regulated by the GacS/GacA two-factor regulatory system, which positively regulates the expression of small RNA molecules*. RpoZ* gene did not regulate the transcription of *rsmX*, *rsmY* and *rsmZ* genes. This was indicating that the effect of *rpoZ* gene on antibiotic 2,4-DAPG production was not regulated by the expression of *rsmX*, *rsmY* and *rsmZ* gene at transcription level, but was regulated by the *rsmA*, which directly repressing the translation of *phlACBD* mRNA and inducing the reduction the production of 2,4-DAPG.

### 3.5. *RpoZ* regulates transcription of *rpoS* gene

**Figure 5:**
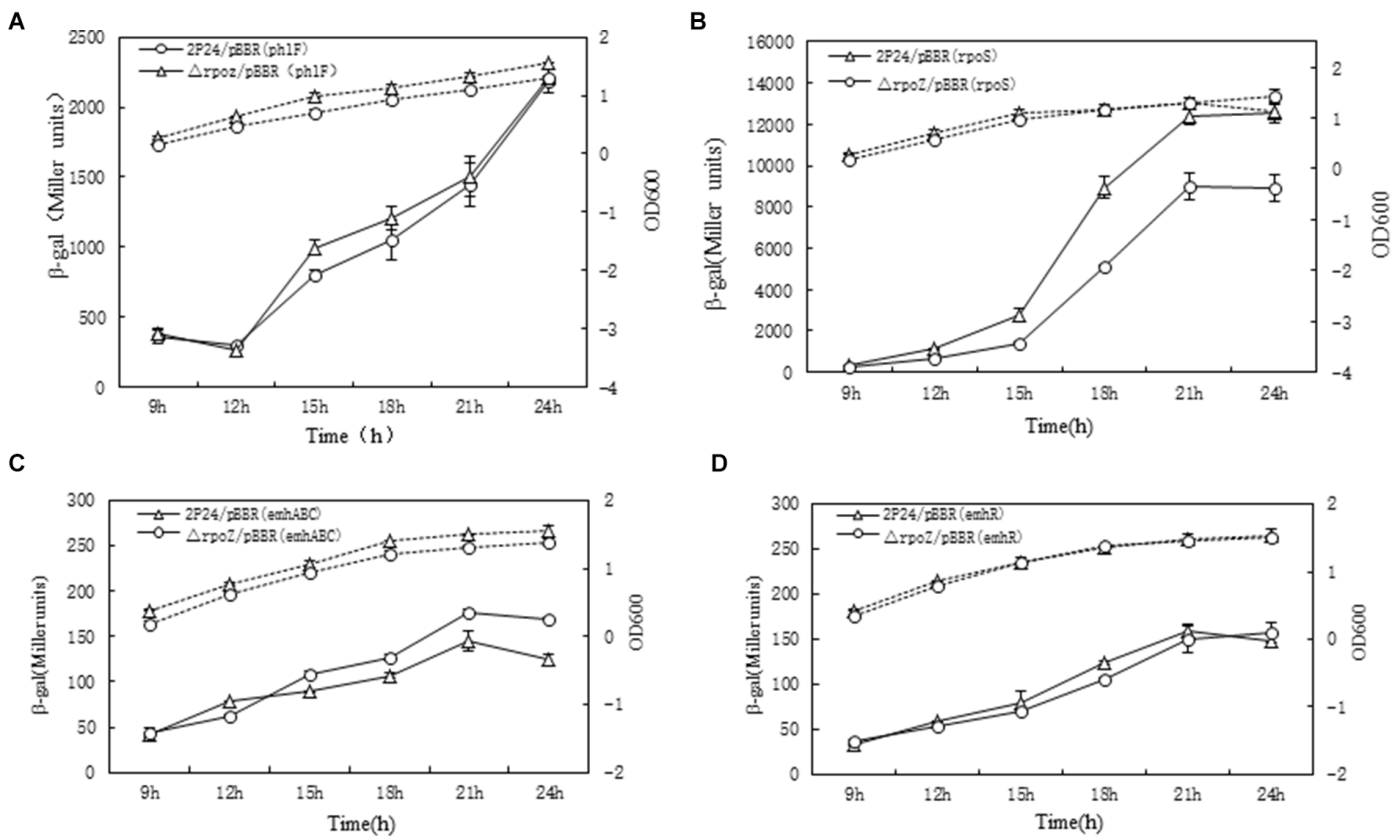
*RpoZ* regulates *phlF, rpoS, emhABC and emhR* expression in *P. fluorescens* 2P24. the β-gal activities in different strains were detected at different time points as shown in **(A–D)**. The β-galactosidase activity to OD_600_ value of each detected strain was shown as the solid lines with Miller units. The dotted lines were growth curve. Each value was calculated by 3 replicates. The values were from at least three independent assays. Three independent experiments were performed and the error bars were calculated.

*PhlF* is an inhibitor of 2,4-DAPG synthesis. By binding to the operon of PhO, PhlF protein can inhibit the binding of its RNA synthase and the region of the *phlA* gene promoter, thus inhibiting the transcription of *phlACBD* gene (Schnider-Keel et al., 2000; Li et al., 2018). However, with the growth of bacteria, a small amount of 2,4-DAPG can bind PhlF protein to counter this inhibition effect, which resulted to synthesize a large amount of 2.4-DAPG. As can be seen from Figure 5A, theβ-galactosidase activity of △rpoZ/pBBR and wild type 2P24/pBBR showed no difference, indicated that *rpoZ* gene had no effect on *phlF* gene transcription. Therefore, it was believed that *rpoZ* did not regulate the expression of *phlF* gene at the transcriptional level in strain 2P24.

*RpoS* is an important regulatory factor in the quiescent phase of bacteria (Hengge-Aronis, 2002), which is regulated by bacteria growth state. And *rpoS* affects the synthesis of secondary metabolites. The *rpoS* gene promoter fusion plasmid rpoS-LacZ was transferred into the wild strain 2P24 and the mutant strain respectively, and the β-galactosidase activity was detected. The results showed that the β-galactosidase activity of the wild-type strain was 1,189 Miller units, and the mutant strain was 800 Miller units. This indicated that *rpoZ* gene positively regulated the expression of *rpoS* at the transcriptional level (Figure 5B). From Figure 5B, we can also see that *rpoS* gene plays a certain role in the growth of bacteria. Before the stable period, the growth of *rpoZ*-deficient strain was slower than that of wild-type strain, but after the stable period, the growth of *rpoZ*-deficient strains was faster than that of wild-type strain.

The multidrug-resistant pump with active bacterial efflux is a system that bacteria can resist starting under adverse environment, by which harmful substances can be excluded from the cell. EmhR-emhABC pumps in *P. fluorescens* 2P24 are typical multi-drug resistant pumps. EmhR is the regulator of EmhABC pump, and the transcription level is negatively regulated by EmhABC. As shown in Figures 5C,D, there was no difference in β-galactosidase activity between mutant △rpoZ/pBBR and wild-type 2P24/pBBR, suggested that *rpoZ* did not regulate the expression of emhABC and emhR genes at the transcriptional level in strain 2P24. The growth curve of culture medium showed that *emhR* gene had no obvious influence on the bacteria growth.

### 3.6. Determination of biocontrol characteristic of *rpoZ* in *Pseudomonas fluorescens* 2P24

**Figure 6:**
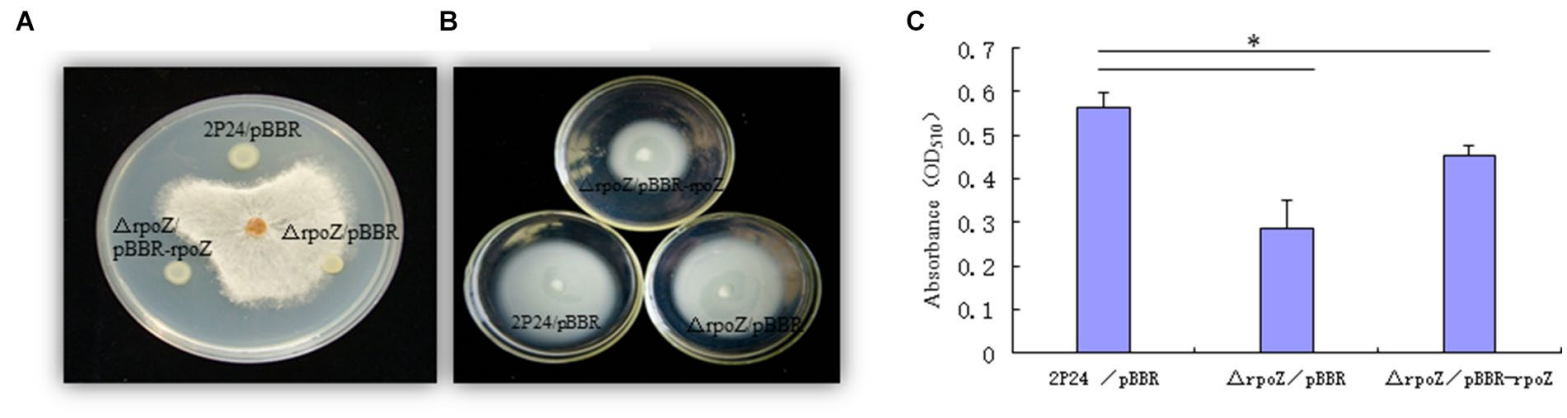
Determination of biocontrol characteristic of *rpoZ* in *P. fluorescens* 2P24. **(A)** Antagonistic ability of *rpoZ* in *P. fluorescens* 2P24 and its derivates against *Rhizoctonia solani* are shown. **(B)** The mobilities of *P. fluorescens* 2P24 and its derivates on medium are shown. **(C)** Regulation of *rpoZ* on biofilm formation. *p < 0.05 by Student’s *t* test.

Previous studies have shown that 2,4-DAPG is the main biocontrol factor in many biocontrol bacteria (Wei and Zhang, 2005; Tian et al., 2010; Zhao et al., 2020). HPLC results showed that the wild strain could produce 2,4-DAPG, while the mutant strain △rpoZ/pBBR produced very little 2,4-DAPG (Figure 6A; Table 1). The antagonism experiment also proved that the mutant strain of *rpoZ* lost the antagonism ability against the *Rhizoctonia solani* in cotton. These results indicated that *rpoZ* had an important antagonistic effect on 2,4-DAPG pathogens. *RpoZ* gene had a significant effect on the swimming of the strain. The swimming of *rpoZ* deletion mutant was significantly slower than that of the wild type (Figure 6B; Table 2).

**Table 1:** Antagonistic ability of *rpoZ* in *Pseudomonas fluorescens* 2P24 and its derivates against *Rhizoctonia solani.*

	Antagonistic zone of no pathogen growth (mm)
2P24/pBBR	8.2 ± 0.2
△rpoZ/pBBR	0
△rpoZ/pBBR-rpoZ	8.0 ± 0.3

Data are averages of three repeat.

**Table 2:** The mobility of *P. fluorescens* 2P24 and its derivates.

Strains	The mobility of bacteria (mm)
2P24/pBBR	65 ± 1.0
△rpoZ/pBBR	40 ± 0.8
△rpoZ/pBBR-rpoZ	62 ± 0.7

Data are averages of three repeat.

Biofilm is a solid structure similar to membrane formed by extracellular polysaccharides and microorganisms on the solid surface. Bacteria in the biofilm form an interactive community and are not free planktic cells (Cvitkvitch et al., 2003). It has been reported that *pcoI* positively regulated the formation of biofilm in *P. fluorescens* 2P24, and *rpoZ* positively regulated the transcription expression of *pcoI* (Wang et al., 2021). Therefore, we tested the regulatory effect of *rpoZ* gene on biofilm. We found that the formation of biofilm in *rpoZ* mutant strains decreased significantly compared with wild-type strain, and the complementary strain also basically recovered the level of wild-type strain (Figure 6C). This indicated that *rpoZ* gene had an effect on biofilm formation.

## 4. Discussion

It was well known that the widely conserved omega subunit encoded by RpoZ is the smallest subunit of *Escherichia coli* RNA polymerase but is dispensable for bacterial growth (Sarkar et al., 2013). *RpoZ* is located upstream of *spoT* and shared a promoter with *spoT*. *SpoT* and *rpoZ* mutant strains showed slower growth. The researchers concluded that the slower growth phenotype of *rpoZ* mutant could be inhibited by *relA*, suggesting that the slower growth was not caused by the loss of *rpoZ*, but was the result of increased (P)ppGpp levels due to changes in polarity (Wendrich et al., 2000). To mimic the strict response, RNAP (with or without ω subunit) was analysed using an *in vitro* fusion transcription system. ω subunit was involved in the regulation of intracellular ppGpp. ω subunit was involved in the regulation of *relA* gene expression. When ω subunit was absent, *relA* transcriptional expression was decreased, thus ppGpp level and mRNA expression were decreased. The insertion mutation of *rpoZ* conferred a slow-growth phenotype when it was introduced into most strains (Gentry and Burgess, 1989). These results suggested that *rpoZ* indirectly regulates the intracellular levels of (P)ppGpp (Gentry and Burgess, 1989). The slower growth phenotype of *rpoZ* mutant wasn’t attributed to the polar of *spoT* in the downstream. The researchers concluded that the slower growth phenotype of *rpoZ* mutant could be inhibited by *relA*, suggesting that the slower growth was not caused by the loss of *rpoZ*. And deletion of ω subunit does not affect the synthesis of (p)ppGpp, and any difference in phenotype observed is possibly due to the reduced binding of (p)ppGpp to RNAP in ∆rpoZ strain (Bhardwaj et al., 2018). But other scientists reported that deletion of ω does not affect the synthesis of (p)ppGpp, and any difference in phenotype observed is possibly due to the reduced binding of (p)ppGpp to RNAP in ∆rpoZ strain (Bhardwaj et al., 2018), So far, ∆rpoZ stain showed slow growth because of the influence of (p)ppGpp, rather than the polar of the *spoT* gene.

To further explore the regulation role of *rpoZ*, in this study, we identified *rpoZ* gene from *P. fluorescens* 2P24 and demonstrated it had positive regulation role in the expression of *phlA* at the transcriptional level, which affected the production of antibiotic 2,4-DAPG, and it resulted in the decreased biocontrol ability in ∆rpoZ stain (Zhou et al., 2005). It has been showed that the *rpoZ* gene was required for antibiotic production and morphological differentiation but is not essential for growth in *Streptomyces kasugaensis* (Kojima et al., 2002; Santos-Beneit et al., 2011). Deletion of the gene *rpoZ* in *Mycobacterium smegmatis* results in reducing growth rate, a change in colony morphology and fragmentation of the beta’ subunit in the enzyme assembly (Mathew et al., 2005). In *rpoZ* mutant, production of actinorhodin,undecylprodigiosin and gray pigment closely associated with spores decreased, especially the expression of actinorhodin and gray pigment was completely inhibited (Santos-Beneit et al., 2011).

Previous studies have shown that PcoI/PcoR QS system in strain *P. fluorescens* 2P24 positively regulates biofilm formation and colonization of root circumference (Yan et al., 2009a,b). *RpoZ* gene positive regulation role in the expression *pcoI* gene at the transcriptional level, which affected the production of the signalling molecule AHL, and *RpoZ* gene positively regulated QS system. Therefore, we proposed that *rpoZ* could also affect colonization ability of root circumference and biofilm formation through QS system, thus affecting biocontrol ability. It reported that ΔrpoZ strain of *E. coli* showed defective biofilm formation only in minimal media and this indicated that ω subunit plays an important role in biofilm formation under stress conditions (Weiss et al., 2017). ΔrpoZ strain in *M. smegmatis* and *S. aureus* is known to be defective in biofilm formation (Mathew et al., 2006; Weiss et al., 2017).

The absence of *RpoZ* leads to a different set of genes being transcribed as seen in *E. coli*. It had an effect on the expression of *rsmA*, but it had no effect on the expression and transcription of untranslated other small RNA (Weiss et al., 2017). *RsmA* and *rsmE* are carbon storage regulatory factors, which can bind to the ribosome binding site RBS of mRNA transcribed by secondary metabolites HCN, 2,4-DAPG and Plt synthesis genes, thus preventing the initiation of translation and regulating the synthesis of these secondary metabolites.

## 5. Conclusion

Using Tn5 mutagenesis, we obtained *rpoZ* mutant in *P. fluorescens* 2P24. This facilitated us to study the *rpoZ* function. Our data showed that *rpoZ* gene was an important regulator of antibiotic 2,4-DAPG and QS system, which is important for investigating the mechanism of biocontrol activity in *P. fluorescens* 2P24.

## Data availability statement

The original contributions presented in the study are included in the article/Supplementary material, further inquiries can be directed to the corresponding author/s.

## Author contributions

MZ, HZ, and BD wrote the manuscript. LZ, XW, and HZ designed the experiments. YW and XW performed the experiments. BD, DW, NL, and QZ analyzed the sequencing data. BD and DW advised on the English language editing. All authors contributed to the article and approved the submitted version.

## Funding

This work was supported by the National Natural Science Foundation of China (grant no. 30860166), Science and Technology Xingmeng project of Inner Mongolia in China (grant no. KJXM-EEDS-2020008), and China Agriculture Research System of MOF and MARA (grant no. CARS-07-C-3).

## Conflict of interest

The authors declare that the research was conducted in the absence of any commercial or financial relationships that could be construed as a potential conflict of interest.

## Publisher’s note

All claims expressed in this article are solely those of the authors and do not necessarily represent those of their affiliated organizations, or those of the publisher, the editors and the reviewers. Any product that may be evaluated in this article, or claim that may be made by its manufacturer, is not guaranteed or endorsed by the publisher.
