# Supplementary material for: RpoZ regulates 2,4-DAPG production and quorum sensing system in Pseudomonas fluorescens 2P24
Source: Front Microbiol. 2023 May 12;14:1160913. doi: 10.3389/fmicb.2023.1160913 (PMC10213339; doi:10.3389/fmicb.2023.1160913)
Supplement: Supplementary file 1 [file Table_1.docx]

**Supplementary table1**

菌株与质粒

Strains and plasmids

| Strains and plasmids | | Characteristics | Source |
| --- | --- | --- | --- |
| Stains | | | |
| *Pseudominas.f luorescens* | | | |
| 2P24 Wild-type，Ap^Ｒ^  Lab collection | | | |
| PM901 | Derivative of 2P24，phlA-lacZ reporter fusion， Ap^Ｒ^ | | 田涛等，2009 |
| 2P24△rpoZ | Derivative of 2P24，*rpoZ* gene in-frame deletion mutant；Ap^Ｒ^ | | This study |
| 2P24△rpoZ-pBBR | Derivative of 2P24，2P24△rpoZ containing pBBR-Gm；Ap^r^；Gm^Ｒ^ | | This study |
| 2P24△rpoZ-rpoZ | Derivative of 2P24，2P24△*rpoZ* containing rpoZ；Ap^Ｒ^；Gm^Ｒ^ | | This study |
| *Escherichia coli* DH5α | F^-^ recA I endA I hsdR I7 deoR thi-I supE44 gyr A96 relA I △（lacZYA-argF）U169 λ^-^（φ80dlacz△M15） | | Sambrook et al.,1989 |
| *Agrobaterium umefaciens* NTL4（pZLR4） | A.tumefaciens NT1 derivative carrying a traG-lacZ reporter fusion，Ap^Ｒ^ | | Cha et al.,1983 |
| Plasmids | | | |
| pBluescript Ⅱ SK+ | Cloning vector，ColE 1 replicon;Ap^Ｒ^ | | Stratagene |
| pRK415 | Broad-host-rang cloning vector; Incp 1 replicon; polylinker of pUC19;Mob^＊^;Ter^Ｒ^ | | Keen，et al. 1988 |
| pBLR | Derivative of pBS，containing intact lacZYA;Km^R^ | | 刘九成等，2010 |
| pBBR | Cloning vector ,Mob; Gm^Ｒ^ | | Lab collection |
| pUTGm | Delivery plasmid for Tn5; R6K relicon; Ap^Ｒ^;Gm^Ｒ^ | | Herrero,et al.1990 |
| pBBR-rpoZ | pBBR containing intact rpoZ gene , Gm^Ｒ^ | | This study |

注：Amp^R^, Cm^R^, Gm^R^, Km^R^, Ter^R^, indicate resistance to amplicilin, chloromycetin, gentamicin, kanamycin and tetracycline, respectively.
