# Supplementary material for: RpoZ regulates 2,4-DAPG production and quorum sensing system in Pseudomonas fluorescens 2P24
Source: Front Microbiol. 2023 May 12;14:1160913. doi: 10.3389/fmicb.2023.1160913 (PMC10213339; doi:10.3389/fmicb.2023.1160913)
Supplement: Supplementary file 2 [file Table_2.docx]

**Table S2 The primers used in this study**

| Primer | Sequuence（5’→3’） | Restrict enzyme |
| --- | --- | --- |
| rpoZ 29729 | ATAGAATTCGGCAACCTCTATGGCACTTCG | *EcoR* I |
| rpoZ 30442 | ATTGGTACCAGTTGACGGGCACGCTTGGTAG | *Kpn* I |
| rpoZ 30518 | ATTGGTACCGAAGGCCTGATGAGCTACGAG | *Kpn* I |
| rpoZ 31424 | ACTAAGCTTGCCGCGCATTTTCTTGTAGATG | *Hind* III |
| rpoz 30059 | ATTAAGCTTACCTGATCATCAACGACGATTTCG | *Hind* III |
| rpoz 30712 | ATAGGTACCGATAAGCGATCGGCGAGGGCGTC | *Kpn* I |
