# Supplementary material for: RpoZ regulates 2,4-DAPG production and quorum sensing system in Pseudomonas fluorescens 2P24
Source: Front Microbiol. 2023 May 12;14:1160913. doi: 10.3389/fmicb.2023.1160913 (PMC10213339; doi:10.3389/fmicb.2023.1160913)
Supplement: Supplementary file 3 [file Table_3.docx]

**Table S3 Mutants with Tn5 insertion and their function**

| Number | Mutant name | Function |
| --- | --- | --- |
| 1 | Aa 2-22 | polyphosphate kinase |
| 2 | Aa2-16 | alginate biosynthesis sensor protein KinB |
| 3 | Aa3-7 | putative regulatory protein of cro/cI family |
| 4 | Aa4-22 | GTP-binding protein TypA |
| 5 | Aa4-16 | TetRfamilytranscriptional regulator |
| 6 | Aa4-29 | DNA-directed RNA polymerase subunit omega |
| 7 | Aa2-39 | ATP-dependent protease ATP-binding subunit ClpX |
| 8 | Aa3-11 | nicotinate phosphoribosyltransferase |
| 9 | S4-11 | putative transport-related membrane protein |
| 10 | N9-4 | oxygen-independent coproporphyrinogen III oxidase |
| 11 | S14-30 | putative peptide synthetase |
| 12 | M25-26 | GTP-binding protein LepA |
| 13 | O32-3 | pyruvate dehydrogenase |
| 14 | T39-1 | conserved hypothetical protein |
| 15 | U28-39 | putative conserved exported protein |
| 16 | N27-24 | lipid A biosynthesis lauroyl acyltransferase |
| 17 | N39-33 | peptide synthase |
| 18 | U35-43 | glutamate-cysteine ligase |
| 19 | C16-5 | putative ubiquinol--cytochrome C reductase, cytochrome C1 |
| 20 | V1-18 | Putative biopolymer transport protein |
| 21 | M24-26 | putative calcium-binding protein |
