# Supplementary figures and images for: RpoZ regulates 2,4-DAPG production and quorum sensing system in Pseudomonas fluorescens 2P24
Source: Front Microbiol. 2023 May 12;14:1160913. doi: 10.3389/fmicb.2023.1160913 (PMC10213339; doi:10.3389/fmicb.2023.1160913)

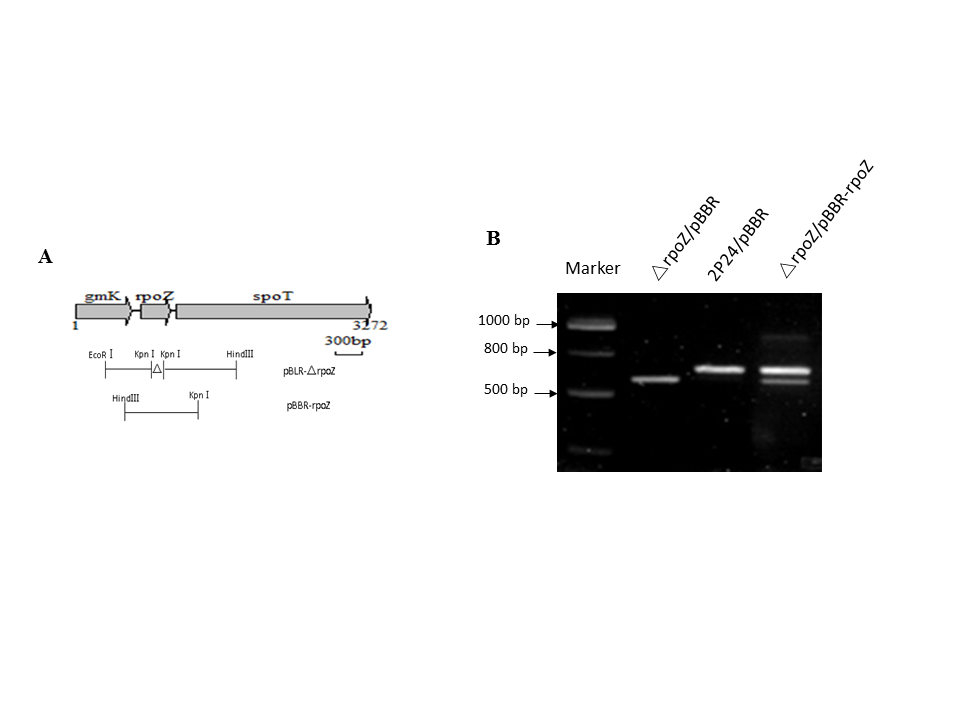

Supplement: Supplementary file 4 [file Image_1.TIF]
